# Implementation of a multimodal strategy via a mobile application to reduce catheter failure in patients with vascular access devices in Spain (CUIDAVEN Study): a pre-post intervention study

**DOI:** 10.1186/s13756-025-01670-y

**Published:** 2025-12-05

**Authors:** Jesús Bujalance-Hoyos, Margarita Enríquez de Luna-Rodríguez, Ana Carmen González-Escobosa, Ana María Oña-González, Silvia Sánchez-Gómez, Antonio Zamudio-Sánchez, Ian Blanco-Mavillard

**Affiliations:** 1 Hospital Regional Universitario de Málaga, 29009 Málaga, Andalusia Spain; 2 Hospital Universitario Costa del Sol, 29603 Marbella, Andalusia Spain; 3 Research Group AA-20 (INVESCUIDA), Instituto de Investigación Biomédica de Málaga y Plataforma en Nanomedicina-IBIMA Plataforma BIONAND, 29590 Málaga, Spain; 4 Research Group BE-23 (Nuevos Horizontes para oncológicos), Instituto de Investigación Biomédica de Málaga y Plataforma en Nanomedicina-IBIMA Plataforma BIONAND, Málaga, Spain; 5 Research Group C-13 (Cronicidad, Dependencia, Cuidados y Servicios Sanitarios), Instituto de Investigación Biomédica de Málaga y Plataforma en Nanomedicina-IBIMA Plataforma BIONAND, Málaga, Spain

**Keywords:** Mobile applications, Catheter-related bloodstream infection, Vascular access devices, Evidence-based practice, Patient safety

## Abstract

**Background:**

Two billion vascular access devices (VADs) are used each year worldwide for the administration of intravenous therapy. Among the most serious complications are catheter-related bloodstream infections (CRBSIs), which increase morbidity and mortality and reduce patients’ quality of life. The aim of this study was to evaluate the impact of implementing a multimodal intervention through a mobile application (CUIDAVEN, Nursing-led Vascular Access Care) on reducing catheter failure and the healthcare costs associated with CRBSIs, while improving nurses’ adherence to best practice recommendations for vascular access care.

**Methods:**

We conducted a quasi-experimental pre-post intervention study without a control group, from April 2019 to August 2022, at the Hospital Regional University of Málaga (Spain). Adult patients requiring VADs (short peripheral intravenous catheters, midlines, peripherally inserted central catheter, and centrally inserted central catheters) for intravenous therapy and capable of using a mobile application were included. Patients with cognitive impairment or in a terminal condition were excluded. The intervention involved the use of the CUIDAVEN mobile application, which provided educational resources, reminders, and monitoring tools. Data were collected during the pre- and post-intervention phases. Variables analysed included sociodemographic characteristics, adherence to good practices, health outcomes, and resource consumption.

**Results:**

A total of 378 patients and 968 VADs were analysed. Catheter failure rate decreased by 19.42% in the post-intervention phase (from 16.95% to 12.24%), with an 80% reduction in CRBSIs (from 2.30% to 0.61%). Mean cost per complication fell from €310.66 to €118.79 (*p*=0.007). Significant improvements were observed in adherence to best practices. Patients also reported increased knowledge and satisfaction with the use of CUIDAVEN.

**Conclusions:**

The implementation of the CUIDAVEN mobile application was associated with a reduction in both infectious and non-infectious complications and improved adherence to recommended practices. Patients perceived greater knowledge and satisfaction, highlighting the potential of digital health tools to empower individuals and improve health outcomes.

**Supplementary Information:**

The online version contains supplementary material available at 10.1186/s13756-025-01670-y.

## Introduction

More than two billion vascular access devices (VADs) are inserted each year worldwide in the context of healthcare delivery [1, 2]. Up to 80% of hospitalised patients are likely to require a VAD during their stay [3], making these devices essential for the administration of intravenous drugs, fluids, blood products, and for delivering renal replacement therapy. However, their use is not without risk, and the burden of complications remains underestimated by many healthcare professionals [4, 5]. Among the most severe complications are catheter-related bloodstream infections (CRBSIs), which are associated with substantial morbidity and mortality, adversely affecting patients’ quality of life and generating considerable human, social, and economic costs [6–8].

Evidence-based measures—such as hand hygiene, aseptic technique during insertion, appropriate catheter maintenance, and timely device removal—are essential to prevent catheter failure and its associated infectious complications [9, 10]. Yet, adherence to these practices remains suboptimal, often due to cognitive and behavioural barriers that hinder sustained improvement in care quality [11]. These shortcomings underline the urgent need for innovative and practical quality improvement strategies that can be embedded into routine clinical practice [12].

Such strategies should incorporate systematic education of all stakeholders, standardised protocols, and timely audit and feedback to support real-time clinical decision-making [13–15]. In this context, a multimodal intervention delivered through a dedicated mobile application may offer a comprehensive solution to improve vascular access care [5]. By supporting adherence to best practice, such tools have the potential to reduce preventable complications, lower healthcare costs, and enhance the overall safety and efficiency of care delivery. Therefore, the primary aim of this study was to assess the impact of implementing a multimodal intervention via a mobile application on catheter failure and catheter-related infection costs, by improving nurses’ adherence to best practice recommendations for vascular access care.

## Methods

### Study design and setting

We conducted a quasi-experimental pre–post intervention study without an equivalent control group, carried out between April 2019 and August 2022 in the same clinical units using identical eligibility criteria and data collection procedures across both phases. The study was conducted at the *Hospital Regional Universitario de Málaga*, a tertiary referral centre within the Andalusian public health system (*Servicio Andaluz de Salud*). The hospital complex comprises three main hospital centres—Hospital General, Hospital Civil, and the Maternal and Child Hospital—along with one High-Resolution Hospital. Altogether, the institution has 1509 inpatient beds distributed across its various hospital facilities and provides comprehensive medical and surgical care to a catchment population of approximately 600000 inhabitants in the province of Málaga. The hospital offers all major specialties, including cardiology, general surgery, neurosurgery, oncology, paediatrics, and psychiatry, among others. This setting allowed for the implementation of the intervention across diverse clinical environments, including both inpatient hospital wards and home-based care through the vascular access specialised team (VAST). This quality-improvement, non-randomized pre–post implementation study was not a clinical trial and therefore did not require trial registration; ethics approval was obtained (PIN-0288-2018).

### Participants

Eligible participants were patients aged between 14 and 99 years who were receiving active intravenous therapy requiring a VAD during hospital admission. Patients were recruited from a range of clinical settings representing both medical and surgical units. Inclusion also required that the patient or their caregiver had the necessary digital literacy to operate a smartphone and use a mobile application. To reduce clinical confounding, only patients who were free from active concurrent infections at the time of vascular access device insertion were included. Exclusion criteria included cognitive impairment, being in the terminal phase of illness, or declining to provide informed consent to participate in the study. We recruited consecutive eligible patients from the same clinical units, providing context-level control of case mix and organisational factors across phases.

### Procedures/Data collection

Data collection was carried out during two distinct phases: the pre-intervention period (4 April 2019 – 18 May 2019) and the post-intervention period (20 December 2021 – 26 august 2022). The intervention itself was implemented between June 2020 and December 2021 across all participating units.

During the pre-intervention phase, data were collected using structured forms, which were subsequently digitised intro a centralised excel database. In the post-intervention, data collection was performed through the web platform integrated into the CUIDAVEN (Nursing-led Vascular Access Care) mobile application. This digital case report form (CRF) ensured standardised and systematic recording of all study variables across wards. Before full implementation, a pilot phase was conducted to ensure the feasibility of the multimodal intervention delivered through the mobile application. This stage allowed for refinement of the intervention and mitigation of potential errors during data collection. In parallel, clinical investigators contacted eligible participants to obtain informed consent. All relevant study documentation was provided, and the mobile application was installed on the participant’s or caregiver’s smartphone to facilitate review of the information sheet and completion of the consent process. Subsequently, data were collected for all VADs used by participants, including short peripheral intravenous catheters (PIVCs), midline catheters, peripherally inserted central catheters (PICCs), centrally inserted central catheters (CICCs), tunnelled dialysis catheters, and subcutaneous ports (PORTs).

Health-related quality of life was evaluated using The EuroQol-5D-5L, which comprises five dimensions: 1. Mobility; 2. Self-care; 3. Usual activities; 4. Pain/Discomfort; and 5. Anxiety/Depression and includes both an index score and a visual analogue scale (VAS). Scores were converted to index values using the validated Spanish tariff, where 1.0 represents perfect health and values below 0.6 are considered indicative of impaired quality of life. VAS values below 50 were interpreted as perceived poor health status. This tool enables comparison across patient groups and the general population, supports longitudinal health status tracking, and informs effectiveness measures in health economic evaluations.

### Variables

The primary endpoint was overall catheter failure (removal due to any complication). Secondary endpoints included subtype-specific complications (phlebitis, infiltration, extravasation, local infection, CRBSI rate, haematoma, pneumothorax, MARSI), adherence to insertion / maintenance recommendations, patient satisfaction, and self-perceived knowledge, and resource use and costs related to VAD complications. We report CRBSI as a proportion of devices removed due to bloodstream infection in this pre–post comparison. Detailed definitions of vascular access devices, catheter-related complications, and diagnostic criteria are provided in Supplementary Appendix S1.

1. *Sociodemographic variables* age, sex, admitting unit, type of VAD, and EuroQol-5D-5L questionnaire.
2. *Variables related to adherence to best practices* hand hygiene, optimal device selection (calibre and insertion site), use of antiseptic agent, verification of proper function, and provision of patient education regarding self-care and maintenance.
3. *Health outcome variables* catheter failure rate and subtype-specific failure rates (e.g., phlebitis, infiltration, extravasation, local infection and CRBSI, haematoma, pneumothorax, and medical adhesive-related skin injuries – MARSI), patient satisfaction with the mobile application, and self-perceived knowledge about self-care.
4. *Resource use and economic variables* number of hospital admissions or delays in discharge due to vascular access complications or adverse events, number of nursing visits for device-related complications, and estimated economic cost of complications and adverse events. Respect to economic analysis, a hospital perspective and in-hospital time horizon were adopted. Direct medical costs included consumables and staff time for device replacement, imaging and laboratory tests, antimicrobial therapy, wound care, and additional bed-days when attributable to VAD complications. Unit costs were obtained from hospital finance schedules (2021–2022) and expressed in euros (2022). Mean cost per complication was compared pre- vs post-intervention.

### Sample size calculation

A total of 4909 patients were admitted to the participating Clinical Management Units during the one-year period, including general surgery (*n*=2308); oncology (*n*=741); haematology (*n*=872); critical care (*n*=418); paediatrics (*n*=220); and VAST (*n*=350). This population served as the institutional reference frame for estimating the required sample size. Assuming a two-sided test with an alpha risk of 0.05 and a beta risk of 0.2 (80% power), and based on previous audits and implementation studies, a reduction in vascular access device–related complications from 40% in the pre-intervention phase to 25% in the post-intervention phase was anticipated. Under these parameters, the minimum required sample size was 152 patients per phase, which was adjusted to 169 per phase (338 in total) to account for a potential 10% attrition rate. A total of 189 patients were recruited in each phase, exceeding the calculated minimum and ensuring sufficient statistical power to detect clinically meaningful differences between groups.

### Intervention

The proposed intervention focused on the development and implementation of the CUIDAVEN mobile application, designed to support the care of VADs. CUIDAVEN is a proprietary, non-commercial application funded by the Andalusian Regional Ministry of Health and developed by the Andalusian School of Public Health, fully compliant with institutional data protection and research ethics requirements.

**Fig. 1:**
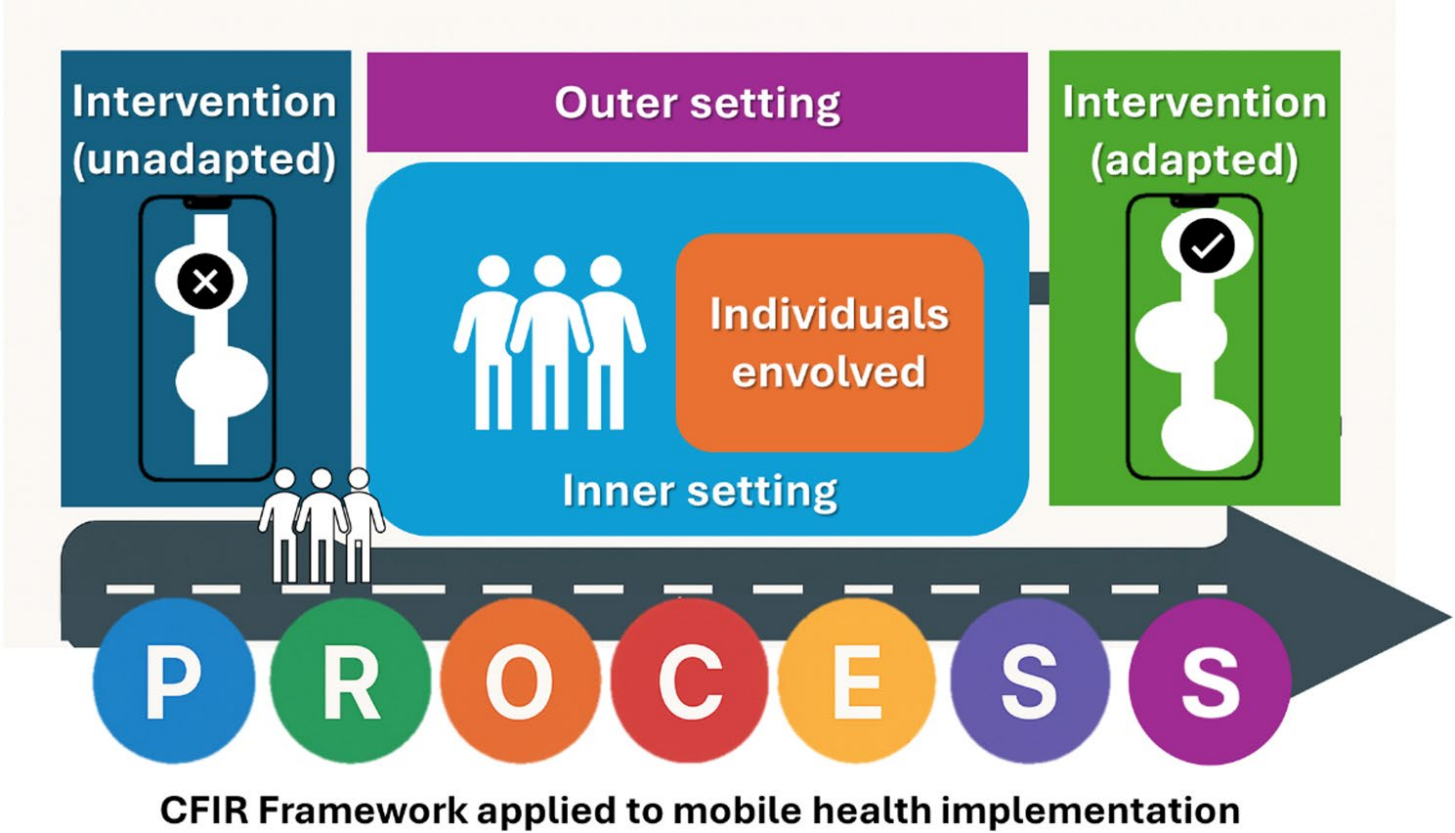
CFIR diagram illustrating the adaptation process of an intervention to the local context

This intervention was based on a multimodal strategy grounded in the Consolidated Framework for Implementation Research (CFIR) [16], which provides a comprehensive structure for understanding the factors and dimensions influencing the implementation of innovations within healthcare settings—namely the intervention itself, the inner and outer settings, the individuals involved, and the implementation process (see Fig. 1).

The interaction between each component of the multimodal intervention and the dimensions of the theoretical framework is detailed below:

#### Intervention characteristics

A mobile application (CUIDAVEN) was developed to provide educational resources, care reminders, and patient follow-up tools. Beyond merely delivering information via videos, posters, or infographics, the intervention also included accredited workshops and clinical training sessions covering insertion, maintenance, management, and removal of VADs. The mobile application’s adaptability was a key feature, enabling personalisation to meet the specific needs of different clinical environments within a tertiary hospital, thus enhancing its acceptability and usability in real-world settings. All content within the mobile application was based on evidence extracted from current clinical practice guidelines, validated by a national expert consensus that tailored each recommendation to the local context. In addition, users could report and share experiences, thereby enriching the knowledge base with real-time, practice-based insights.

#### Outer setting

The intervention was funded by the Andalusian Regional Ministry of Health and Consumer Affairs and developed by the Andalusian School of Public Health. It was fully aligned with health policies, data privacy regulations, and ethical standards for research. These synergies fostered collaboration among stakeholders in the fields of vascular access and health technology, ensuring that the application met standards of quality, effectiveness, and accessibility for healthcare professionals.

#### Inner setting and individual characteristics

Support from institutional leaders was sought to formally present the intervention. Educational sessions and workshops were delivered to promote a culture of continuous improvement and the adoption of best practices in VAD care. Implementation plans were developed to integrate the mobile application into healthcare professionals’ daily routines, establishing specific moments for use and review—particularly upon catheter insertion—to support patient education and compliance with recommended practices. At those times, patients were introduced to the mobile application via an informational leaflet with a QR code. Another important aspect was the clinical follow-up of patients through the CUIDAVEN-associated web platform, where all clinical data were recorded during the post-intervention phase. Patients carried anonymised information about their catheter on their own mobile devices.

#### Implementation process

A structured implementation plan was developed, including timelines, key milestones, and unit-specific leadership roles. This plan was disseminated to all stakeholders to ensure shared understanding of the process. Systematic monitoring of indicators was conducted by the VAST, who were responsible for leading change and driving process improvement. Continuous feedback mechanisms were established to facilitate routine change and reinforce the intervention components.

CUIDAVEN comprises two complementary interfaces: (1) a clinician-facing module with point-of-care checklists and videos, aseptic insertion indications, maintenance reminders (e.g., dressing change, patency checks), and links to unit protocols; and (2) a patient-facing module with plain-language education, symptom checkers (e.g., redness, pain, discharge), and action requests (e.g., “if you notice purulent exudate or fever, notify your nurse immediately”). Push notifications are timed to insertion and expected maintenance intervals. A web dashboard allowed the VAST to monitor adherence indicators and provide feedback. The flow of use across pre- and post-intervention phases and user roles is shown in Supplementary appendix S2.

In parallel with the digital deployment, full coverage of training and reinforcement activities was achieved. All patients (100%) and healthcare professionals involved received structured, face-to-face education sessions on vascular access device care, infection prevention, and the use of the CUIDAVEN tools. Education was continuously reinforced during each clinical encounter by vascular access nurses, supported by micro-meetings and small-group workshops focused

### Statistical analysis

Data obtained from the export of selected variables for the CUIDAVEN study were stored in an anonymised database created in a Microsoft Excel spreadsheet (Office 365), which was subsequently cleaned and analysed using IBM SPSS Statistics version 25.

Descriptive statistics were used to summarise the variables. Frequencies and percentages were calculated for categorical variables. Quantitative variables were expressed as arithmetic mean ± standard deviation (for normally distributed data) or as median and interquartile range (for non-normally distributed data). Comparisons between categorical variables were performed using the Chi-square test or Fisher’s exact test, as appropriate. For normally distributed continuous variables, parametric tests were used: Student’s t-test for comparisons between two groups, and analysis of variance (ANOVA) for comparisons involving more than two groups. For non-normally distributed variables, non-parametric tests were applied: the Wilcoxon rank-sum test for two-group comparisons, and the Kruskal–Wallis H test for more than two groups. To assess the magnitude of changes between the pre- and post-intervention phases, absolute risk differences (ARDs) were calculated with 95% confidence intervals (95% CI) using Newcombe’s method for independent proportions (Wilson score). Relative risks (RRs) and their corresponding 95% CIs were estimated to quantify the relative improvement associated with the intervention. All statistical tests were interpreted alongside effect size estimates to emphasize the clinical as well as statistical significance of the findings. Cost analysis was conducted to determine the economic impact between the pre- and post-intervention groups. Two-sided p-value <0.05 was considered statistically significant.

## Results

A total of 378 patients were included in the study, with an equal number of participants in the pre- and post-intervention phases (*n*=189 per group). The mean age was approximately 45 years in both phases, with no significant differences observed (45.94 ±21.6 vs 45.17 ±20.99; *p*=0.72). Regarding gender, a slightly higher proportion of males was observed in the post-intervention phase, approaching but not reaching statistical significance (52.38% in the pre-intervention vs 57.14% in the post-intervention phase; *p*=0.05). Hospital units were well represented in both phases, particularly oncology, haematology, and general surgery. No significant differences were found between groups in terms of hospital unit distribution (*p*=0.11). Regarding the EuroQol-5D-5L quality of life questionnaire, no relevant differences were found in any of the assessed dimensions (mobility, self-care, usual activities, pain/discomfort, and anxiety), nor in the EQ-5D index or the visual analogue scale, indicating adequate comparability between groups at baseline. During both study phases, a total of 968 VADs were analysed (478 in the pre-intervention and 490 in the post-intervention phase). The most frequently used VAD was the short PIVC, accounting for 47.63% of all devices—47.07% and 48.2% in each respective phase—followed by PICCs and midlines. No significant differences were observed in the distribution of VAD types between phases (*p*=0.18). All sociodemographic characteristics and quality-of-life outcomes are detailed in Table 1.

**Table 1:** Sociodemographic Characteristics and EuroQoL-5D-5L Questionnaire Results

Variable	Pre-intervention phase	Post-intervention phase	*p*-value
Total patients (n) Age, mean (SD) Sex, n (%) Female, n (%) Male, n (%)	189 45.94 (21.6) 189 (100) 90 (47.61) 99 (52.38)	189 45.17 (20.99) 189 (100) 81 (42.85) 108 (57.14)	0.72 0.05
Unit of admission, n (%) Paediatric, n (%) Surgery, n (%) Oncology, n (%) Haematology, n (%) Nephrology, n (%) Vascular access specialised team, n (%)	189 (100) 24 (12.7) 35 (18.5) 43 (22.8) 39 (20.6) 19 (10.1) 29 (15.3)	189 (100) 24 (12.7) 20 (10.6) 54 (28.6) 36 (19.0) 13 (6.9) 42 (22.2)	0.11
EUROQOL-5D-5L DOMAINS			
1. Mobility Independent (no problems) Dependent (problems)	123 (65.08) 66 (34.92)	127 (67.2) 62 (32.8)	0.66
2. Self-care Independent (no problems) Dependent (problems)	106 (56.08) 83 (43.92)	111 (58.73) 78 (41.27)	0.63
3. Usual activities Independent (no problems) Dependent (problems)	78 (41.27) 111 (58.73)	79 (41.8) 110 (58.2)	0.91
4. Pain / Discomfort No Yes	76 (40.21) 113 (59.79)	79 (41.8) 110 (58.2)	0.75
5. Anxiety No Yes	99 (52.38) 90 (47.62)	103 (54.5) 86 (45.5)	0.68
Visual analogue score, mean (SD)	62.75	63.76	0.199
EQ-5D-5L Index, mean (SD)	0.42 (0.21)	0.44 (0.19)	0.542
Severity index, mean (SD)	63.68 (13.89)	65,21 (11,81)	
VASCULAR ACCESS DEVICES USED			
Vascular access device type, total Short PIVC, n (%) Midline catheter, n (%) PICC, n (%) CICC, n (%) FICC, n (%) Tunnelled catheter, n (%) Subcutaneous port, n (%)	478 225 (47.07) 59 (12.34) 87 (18.2) 33 (6.9) 4 (0.83) 20 (4.18) 50 (10.41)	490 236 (48.2) 67 (13.7) 90 (18.4) 15 (3.1) 2 (0.4) 22 (4.5) 58 (11.8)	0.18

*PIVC: peripheral intravenous catheter; PICC: Peripherally Inserted Central Catheter; CICC: Centrally Inserted Central Catheter; FICC: Femorally Inserted Central Catheter*

Regarding patient health outcomes related to VADs, the overall catheter failure rate was 14.56% (141 events), with a significant 19.42% reduction in the post-intervention phase (16.95% vs 12.24%; RR 0.72 [95% CI, 0.53–0.98]; *p*=0.0038). The most frequent type of catheter failure was phlebitis, affecting 9.3% of all VADs, with no statistically significant differences between phases (10.25% vs 8.37%; RR 0.82 [95% CI, 0.55–1.21]; *p*=0.28). Catheter-related infections showed a global rate of 1.45% (14 infectious events) across the study period, specifically CRBSIs with a significant 80% reduction observed in the post-intervention phase (2.09% vs 0.41%; RR 0.20 [95% CI, 0.04–0.89]; *p*=0.018). Other complications, such as infiltration, also showed notable improvement, with rates decreasing in the post-intervention phase (2.30% vs 0.61%; RR 0.27 [95% CI, 0.07–0.95]; *p*=0.02). All health outcomes related intravenous therapy are presented in Table 2.

**Table 2:** Health outcomes related to catheter failure and complication subtypes.

Variable	Pre-intervention phase	Post-intervention phase	Risk relative Post vs Pre [95% CI]	Absolute risk difference (Post – Pre) [95% CI]	*p*-value
Catheter failure, n (%) Uncomplicated removal, n (%)	81 (16.95) 397 (83.05)	60 (12.24) 430 (87.76)	0.72 [0.53–0.98]	–4.70pp [–9.17 to –0.25]	0.038
Phlebitis, n (%) Grade 1, n (%) Grade 2, n (%) Grade 3, n (%) Grade 4, n (%) Grade 5, n (%)	49 (10.25) 37 (7.74) 8 (1.67) 4 (0.84) 0 0	41 (8.37) 28 (5.71) 10 (2.04) 2 (0.41) 0 1 (0.20)	0.82 [0.55–1.21]	–1.88pp [–5.60 to 1.80]	0.28
Infiltration, n (%)	11 (2.30)	3 (0.61)	0.27 [0.07–0.95]	–1.69pp [–3.51 to –0.14]	0.02
Extravasations, n (%)	6 (1.26)	2 (0.41)	0.33 [0.07–1.60]	–0.85 pp [–2.33 to 0.42]	0.053
Occlusion, n (%)	1 (0.21)	4 (0.82)	3.90 [0.44–34.78]	+0.61 pp [–0.29 to 1.50]	0.19
Catheter-related infection, n (%) Exit-site infection, n (%) CRBSI, n (%)	11 (2.30) 1 (0.21) 10 (2.09)	3 (0.61) 1 (0.20) 2 (0.41)	0.27 [0.07–0.95] 0.98 [0.06–15.55] 0.20 [0.04–0.89]	–1.69 pp [–3.20 to –0.18] –0.01 pp [–0.58 to 0.57] –1.68 pp [–3.42 to –0.25]	0.02 0.98 0.018
Hematoma, n (%)	1 (0.21)	7 (1.43)	6.83 [0.84–55.29]	+1.22 pp [0.01 to 2.72]	0.03
MARSI, n (%)	2 (0.42)	2 (0.41)	0.98 [0.14–6.90]	–0.01 pp [–1.14 to 1.10]	1
Pneumothorax, n (%)	0	2 (0.41)	4.88 [0.23–101.34] *	+0.41 pp [–0.44 to 1.48]	0.16

*CRBSI: Catheter-related bloodstream infection; MARSI: Medical adhesive-related skin injury; pp: percentage points*

^*^ For outcomes with zero events, risk ratios and CIs were computed using the Haldane–Anscombe correction.

Adherence to recommended practices during VAD insertion significantly improved across all evaluated indicators in the post-intervention phase. Specifically, hand hygiene compliance increased from 75.94% to 86.53% (RR 1.11 [95% CI, 1.06–1.22]; *p*<0.001); optimal selection of Short PIVC improved from 23.11% to 42.37% (RR 1.83 [95% CI, 1.38–2.41]; p<0.001); the use of alcoholic chlorhexidine in Short PIVC increased from 33.78% to 77.11% (RR 2.28 [95% CI, 1.86–2.80]; *p*<0.001) and in central devices from 78.95% to 85.03% (RR 1.08 [95% CI, 0.96–1.21]; *p*=0.125). Functional verification of the catheter improved for all devices (from 23.56% to 42.37% for Short PIVC and from 77.73% to 96.25% for other devices; both *p*<0.001). Patient education rates also more than doubled in the post-intervention phase (19.55% to 42.37%; (RR 2.17 [95% CI, 1.59–2.97]; *p*<0.001). All data related to adherence is shown in Table 3.

**Table 3:** Adherence to recommendations for VAD insertion

Recommendations During VAD Insertion	Pre-intervention phase	Post-intervention phase	Risk relative Post vs Pre [95% CI]	Absolute risk difference (Post – Pre) [95% CI]	*p*-value
Total VADs inserted	478	490	**–**	**–**	**–**
Hand hygiene before insertion, n (%) PIVC, n (%) Midline, n (%) PICC, n (%) CICC, n (%) FICC, n (%) Tunnelled catheter, n (%) Subcutaneous port, n (%)	363 (75.94) 167 (74.22) 57 (96.61) 86 (98.85) 28 (84.85) 4 (100) 14 (70) 7 (14.0)	424 (86.53) 215 (91.1) 67 (100) 90 (100) 15 (100) 2 (100) 20 (90.91) 15 (25.86)	1.14 [1.06–1.22] 1.23 [1.12–1.35] 1.04 [0.98–1.09] 1.01 [0.97–1.05] 1.18 [1.00–1.40] 1.00 [0.69–1.44] 1.30 [1.00–1.70] 1.85 [0.87–3.90]	+10.59 pp [3.67 to 17.36] +16.88 pp [7.28 to 25.97] +3.39 pp [−4.49 to 11.54] +1.15 pp [−3.89 to 6.23] +15.15 pp [−1.79 to 31.26] 0.00 pp [−36.92 to 36.92] +20.91 pp [0.66 to 39.24] +11.86 pp [0.28 to 23.16]	<0.001 <0.001 0.217 0.491 0.167 1 0.123 0.127
Optimal PIVC selection (forearm site,20-22G), n (%)	52 (23.11)	100 (42.37)	1.83 [1.38–2.41]	+19.26 pp [10.76 to 27.63]	<0.001
Use of alcoholic Chlorhexidine PIVC, n (%) Midline, PICC, CICC y FICC, n (%)	76 (33.78) 150 (78.95)	182 (77.11) 159 (85.03)	2.28 [1.86–2.80] 1.08 [0.96–1.21]	+43.34 pp [34.67 to 51.44] +6.08 pp [−4.93 to 16.83]	<0.001 0.125
Catheter functionality verified after insertion PIVC, n (%) Midline, PICC, CICC y FICC, n (%)	53 (23.56) 150 (77.73)	100 (42.37) 180 (96.25)	1.80 [1.34–2.43] 1.24 [1.14–1.35]	+18.82 pp [6.72 to 30.27] +18.54 pp [9.46 to 26.83]	<0.001 <0.001
PIVC health education provided, n (%)	44 (19.55)	100 (42.37)	2.17 [1.59 – 2.97]	+22.82 pp [11.01 to 33.85]	<0.001

*PIVC: peripheral intravenous catheter; PICC: Peripherally Inserted Central Catheter; CICC: Centrally Inserted Central Catheter; FICC: Femorally Inserted Central Catheter*

Patient satisfaction with the care process also improved in nearly all items assessed after the intervention phase. Specifically, significant improvements were observed in overall satisfaction (*p*=0.023); perceived quality of information provided (*p*=0.006); understanding of care procedures (p=0.001); perception of having tools for self-care (*p*=0.001); and feeling of safety in handling the VAD (*p*<0.001). However, despite improvement in communication and accessibility scores after the intervention (4.63 to 4.72), the difference was not statistically significant (*p*=0.251). Full satisfaction findings are provided in Table 4.

**Table 4:** Patient satisfaction outcomes among participants (5-point Likert scale)

Variables	Pre-intervention phase (Likert 1-5)	Post-intervention phase (Likert 1-5)	*p*-value
Satisfaction with the information received about care, mean (SD)	4.21 (0.81)	4.44 (0.49)	0.023
Perceived level of information about vascular access care, mean (SD)	3.95 (0.65)	4.22 (0.66)	0.006
Did you have access to information and tools for VAD self-care? mean (SD)	2.16 (1.12)	2.72 (1.49)	0.001
Communication and accessibility with nursing staff, mean (SD)	4.63 (0.69)	4.72 (0.60)	0.251
Did you feel confident managing your VAD? mean (SD)	3.85 (0.91)	4.23 (0.89)	<0.001

*VAD: Vascular Access Device*

The evaluation of patients’ self-perceived knowledge levels, assessed through Nursing Outcomes Classification indicators, showed a significant improvement in the post-intervention phase compared to the pre-intervention phase. Specifically, patients demonstrated increased understanding regarding the purpose of their VAD (mean score 2.73 vs 4.11; *p*<0.001), recognition of signs and symptoms of complications (2.39 vs 3.98; p<0.001), appropriate self-care and hygiene measures (2.55 vs 4.05; *p*<0.001), procedures for contacting healthcare professionals in case of adverse events (2.22 vs 3.84; p<0.001), and overall confidence in managing the device independently at home (2.47 vs 4.09; *p*<0.001). These statistically significant findings confirm the effectiveness of the CUIDAVEN mobile application in enhancing patient empowerment and promoting safe self-management of VADs (Table 5).

**Table 5:** Patients’ Knowledge Level on Self-care According to Nursing Outcomes Classification

Nursing outcome classification (Code)	Pre-intervention phase (Likert 1-5)	Post-intervention phase (Likert 1-5)	*p*-value
Signs and symptoms of infection (184204), mean (SD)	3.55 (0.83)	4.02 (0.8)	0.02
Importance of hand hygiene (184207), mean (SD)	4.16 (0.78)	4.67 (0.35)	<0.001
Correct use of equipment (181404), mean (SD)	4.02 (0.88)	4.51 (0.52)	<0.001
Appropriate care of equipment (181407), mean (SD)	3.73 (1.1)	4.29 (0.67)	0.02
Recognises personal risk factors (190201), mean (SD)	3.99 (0.91)	4.49 (0.71)	<0.001

The mean cost associated with the treatment of VAD-related complications during the pre-intervention phase was €310.66, compared to €118.79 in the post-intervention phase—a statistically significant reduction (*p*=0.007). This cost difference was due to a higher number of complications and catheter failures in the pre-intervention group compared to the post-intervention group (81 vs 60; *p*=0.038), and an increased number of patients with prolonged hospital stays in the pre-intervention phase (33 days vs 12 days; *p*=0.252). All economic impact data is shown in Table 6.

**Table 6:** Resource consumption and economic impact of the CUIDAVEN app

Variables	Pre-intervention phase	Post-intervention phase	*p*-value
Patients requiring extended hospital stay due to a complication Cumulative extra hospital days	5 33	2 12	0.252
Patients with complications related to catheter failure, n (%)	81 (16.95)	60 (12.24)	0.038
Cost associated with the treatment of complications			
Cost associated with the treatment of complications in euros, mean (SD) Non-infectious complications Infectious complications Pneumothorax-related complications	€310.66 (1204.22) €9.48 (17.33) €4838.43 (949.56) Not reported	€118.79 (722.14) €18.54 (38.42) €49997.69 (1516.14) €3247	0.007 0.223 0.114 1

## Discussion

To the best of our knowledge, this is the first study conducted in Spain to evaluate the implementation of a multimodal strategy through the development of a mobile application specifically designed to enhance the quality of care for VADs and patient safety during hospital admission. Our intervention, grounded in the CFIR, enabled the adaptation of all actions and evidence to both internal and external contexts, offering real-time information accessible via the clinician’s and/or patient’s smartphone.

Among the most notable findings, we observed a significant reduction in CRBSIs, as well as a decrease in associated economic burden and resource consumption for their treatment, alongside improved adherence to recommended care practices for VADs. In the post-intervention phase, users also reported higher perceived knowledge regarding their self-care, particularly in relation to the management and maintenance of their vascular access following completion of intravenous therapy. High levels of satisfaction with the mobile application were recorded, highlighting the potential of such tools to improve health outcomes and empower patients requiring intravenous treatment [17, 18].

The overall VAD failure rate observed in our study was nearly 15%, with a 28% reduction in complications during the post-intervention phase. When compared to international studies, our results are consistent with the reported reduction in complication rates [19–21]. Regarding CRBSI rates, our intervention led to an 80% reduction in infectious events compared to the pre-intervention phase. CRBSIs are one of the most serious infectious complications, carrying substantial human, social, and economic consequences [22, 23]. These infections are an independent cause of hospital morbidity and mortality, with each episode potentially increasing hospital stay by 10 to 20 days and healthcare costs by between US$4000 and US$56000, depending on severity [24].

Our intervention proved particularly beneficial in preventing infectious complications among oncology and haematology patients, who, due to their underlying conditions, are more exposed to risk factors and thus more vulnerable to infection and mortality from these causes [25–28]. The economic savings resulting from our intervention are among the most significant findings of this study. We observed a substantial reduction in resource consumption for the treatment of CRBSIs, which are both costly and serious complications. However, we also noted an increase in non-infectious complications during the post-intervention phase, particularly catheter obstructions in long-term devices, which contributed to increased costs in these specific cases. Despite this, the overall cost balance demonstrated clear optimisation of resources and a positive impact on healthcare expenditure for these patients.

We recognise that the effectiveness of this technology in preventing infectious and non-infectious complications was not solely dependent on the implementation process itself, but also on the contextual factors specific to our institution [29, 30], where a dedicated VAST plays a leading role in VAD-related care. This team oversaw the monitoring and follow-up of patients and devices throughout the study. These findings support the critical role of such teams in leading quality improvement initiatives, and the need for implementation strategies that account for interpersonal behaviours and sociocultural environments influencing clinical decision-making. In this capacity, VASTs may act as knowledge brokers, helping to identify and mitigate systemic errors in vascular access care and preventing exposure to non-evidence-based or unnecessary practices [31].

Having access to best-practice knowledge directly via a mobile phone—including reminders, alerts, interactive guidelines, protocols, infographics, posters, presentations, and explanatory videos—provided a clinical environment underpinned by the most robust available evidence, applied to improving VAD care [21]. From a patient safety and quality-of-care perspective, healthcare systems should prioritise the implementation of integrated technologies within routine clinical practice, fostering a deeper understanding of how healthcare professionals make decisions [5, 32]. Our intervention led to a significant improvement in adherence to best practice recommendations, including hand hygiene, optimal device selection for intravenous therapy, use of alcoholic chlorhexidine during insertion, verification of correct catheter function post-insertion, and patient health literacy for self-care. These practices are recognised as highly relevant and feasible for implementation [4, 33].

During the implementation of our multimodal strategy, mechanisms were embedded to translate knowledge into shared decision-making among all stakeholders. This approach not only prioritised the selection of high-quality evidence but also considered interdisciplinary consensus and user input for local adaptation. These strategies triggered mechanisms that enhanced nurses’ decision-making, contributing to a culture that supports clinical safety improvement [34, 35]. Nonetheless, we observed that more than half of the patients with short peripheral catheters had not received education on VAD self-care. This highlights an ongoing gap in patient knowledge, in line with international findings suggesting that more than half of hospitalised patients are unaware of what a catheter is [11]. Mobile health literacy interventions may serve as key tools to empower patients in their self-care, enabling earlier detection of symptoms and signs that could prevent catheter failure or more serious infectious complications [36–38].

We believe that our study has had a positive impact in ensuring high-quality, safe vascular access care, with an eye towards full implementation and sustainability of the intervention not only in the study units but throughout our institution. This is feasible given that the mobile application is free and openly accessible for download across multiple platforms, and thus its benefits may extend far beyond the observed results. Furthermore, these findings contribute to the growing body of literature on technological tools in nursing care, offering evidence of their effectiveness and promoting integration of new technologies into clinical practice. While the findings suggest potential effectiveness and feasibility, they should be interpreted within the methodological boundaries of a pre-post design. Nevertheless, they underscore the opportunity for future hospitalised patient to receive better care through the use of CUIDAVEN, catalysing change among healthcare professionals delivering such care.

However, certain methodological limitations must be considered when interpreting these results. This was a pre-post intervention study evaluating the implementation of a mobile application. Both phases recruited patients from the same clinical units, which mitigates between-unit differences in case mix and organisational practices, thereby providing partial control of contextual variability. Nevertheless, as a non-randomised design, unmeasured clinical variables such as comorbidities could not be statistically adjusted for and may have influenced the outcomes. It is important to note that the effectiveness of the intervention may have been influenced by the specific context of our institution, which may limit generalisability to other settings. Nevertheless, we support the broader implementation of this strategy in healthcare systems. Additionally, pre-existing gender differences between control and intervention groups must be acknowledged, as these may confound interpretation; for example, women tend to experience more complications in surgical contexts. Therefore, results should be interpreted with caution, particularly when differences reach borderline statistical values (e.g. *p*=0.05), which do not strictly meet the predefined threshold of significance (*p*<0.05). We thus caution against attributing all observed differences solely to the intervention, as gender and other unmeasured factors may play an important role in the generalisability of results. This limitation is inherent to non-randomised designs such as ours, where potential confounding cannot be entirely excluded. In addition, the study spanned pandemic phases (2019–2022), which may have affected staffing, device selection practices, and surveillance intensity. While the pre–post design mitigates some time-varying effects by comparing within-institution phases, residual confounding related to pandemic workload and case-mix cannot be excluded. Finaly, the inclusion criterion of basic digital literacy for patients or caregivers may have limited the representativeness of the study population, particularly among older adult patients or those with reduced access to smartphones or internet connectivity. This potential selection bias may restrict the generalizability of our findings to populations with higher technological access and digital competence. As digital tools become increasingly integrated into patient safety initiatives, ensuring equitable access and support for users with limited digital literacy remains a major challenge for the sustainable implementation of such interventions.

## Conclusion

The implementation of a multimodal intervention through a mobile application proved effective in reducing VAD failure and the healthcare costs associated with CRBSIs. Additionally, the intervention led to significant improvements in adherence to best practice guidelines, patient satisfaction, and patient knowledge of self-care. The adoption of CUIDAVEN in healthcare organisations may foster a culture of continuous improvement and clinical excellence led by nurses, while helping to reduce the variability of care practices that are potentially harmful or unnecessary for patient health.

## Supplementary Information

Below is the link to the electronic supplementary material.

Supplementary Material 1

## Data Availability

The datasets supporting the findings of this study are available from the corresponding author and JB-H, upon reasonable request.

## Abbreviations

VAD: Vascular access device
VAST: Vascular access specialised team
PIVC: Peripheral intravenous catheter
PICC: Peripherally inserted central catheter
CICC: Centrally inserted central catheter
MARSI: Medical adhesive-related skin injury
