# Supplementary material for: Implementation of a multimodal strategy via a mobile application to reduce catheter failure in patients with vascular access devices in Spain (CUIDAVEN Study): a pre-post intervention study
Source: Antimicrob Resist Infect Control. 2025 Dec 5;15:5. doi: 10.1186/s13756-025-01670-y (PMC12797837; doi:10.1186/s13756-025-01670-y)

**Supplementary Appendix S1. Operational Definitions**

1. **Vascular access device types:**

**Short-PIVC:** A peripheral intravenous catheter of ≤6.4 cm in length, inserted into a peripheral vein;

**Micromidline:** A short peripheral catheter (6–10 cm) inserted into a deep peripheral vein (usually the basilic or brachial) and advanced near the axillary vein;

**Midline:** A peripheral catheter usually 10–20 cm in length, inserted into a large vein of the upper arm and advanced to the axillary vein, but not entering the central venous circulation;

**PICC:** A central venous access device inserted through a peripheral vein (basilic, brachial, or cephalic) and advanced until the catheter tip resides in the lower third of the superior vena cava;

**CVC:** A central catheter inserted via direct puncture of a central vein (e.g., subclavian, jugular, or femoral) with the tip located in the superior or inferior vena cava;

**Tunneled Central Catheter:** A long-term central catheter inserted into a central vein (commonly the internal jugular or subclavian), tunneled subcutaneously before entering the vein, and equipped with a cuff to reduce infection risk;

**Port:** A totally implantable central venous access device consisting of a reservoir connected to a catheter, surgically placed under the skin and inserted into a central vein.

1. **Vascular access device-related complications:**

**Catheter failure** was defined as the unplanned removal of a vascular access device (VAD) before completion of therapy due to any mechanical, chemical, or infectious complication that impaired its function or safety. Catheter failure is formed by:

**Phlebitis** refers to inflammation of the vein wall, typically characterized by pain, erythema, swelling, and palpable venous cord, and may result from mechanical irritation, chemical irritation, or infection;

**Infiltration** denotes the inadvertent leakage of non-vesicant solution or medication into the surrounding tissue, leading to local edema, discomfort, or blanching of the skin;

**Extravasation** is the accidental administration of vesicant or irritant substances into surrounding tissues, potentially causing pain, inflammation, blistering, or tissue necrosis;

**Occlusion or obstruction** is defined as the partial or complete blockage of the catheter lumen that prevents or restricts fluid infusion or blood withdrawal, often related to thrombotic or precipitate formation;

**Accidental removal** refers to the unintended dislodgement or extraction of the catheter by the patient or healthcare personnel before the planned end of therapy;

**Catheter-related infection** includes both local and systemic events. Exit-site infection is defined as erythema, induration, or purulent drainage within 2 cm of the catheter exit site, whereas CRBSI is diagnosed when the same organism is recovered from blood cultures drawn from the catheter and a peripheral vein, accompanied by clinical signs of infection. CRBSI was defined according to standard criteria (e.g., IDSA/ECDC): compatible clinical picture plus laboratory confirmation (same organism from catheter tip and peripheral blood, or differential time to positivity, favouring catheter-drawn blood), with no alternative source identified. Local infection refers to exit-site or tunnel infection without bloodstream involvement;

**Hematoma** corresponds to localized bleeding or blood accumulation in the subcutaneous tissue at or around the catheter insertion site, often due to vessel trauma during insertion or removal;

**MARSI** refers to any skin damage, such as erythema, stripping, or blistering, persisting for more than 30 minutes after adhesive removal, caused by the use of dressings or securement devices;

**Pneumothorax** may occur as a mechanical complication during the insertion of central venous catheters when air inadvertently enters the pleural space, leading to partial or total lung collapse.

**Supplementary Appendix S2. Workflow Diagram of CUIDAVEN Implementation Across Study Phases and User Roles**


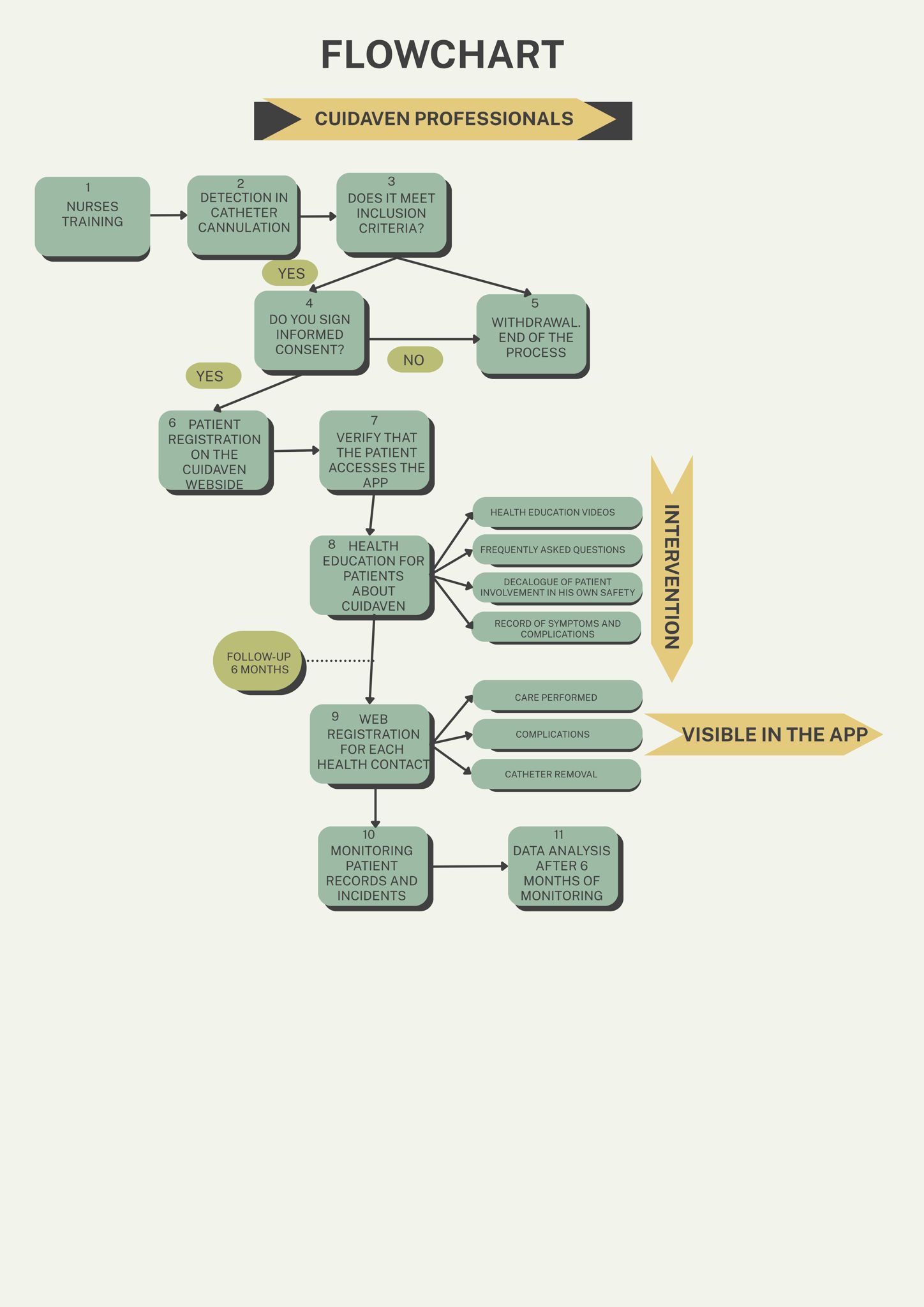

Supplement: Supplementary file 1 — Supplementary Material 1 [file 13756_2025_1670_MOESM1_ESM.docx]
